# Supplementary material for: Comparative multiomics analysis of cell physiological state after culture in a basket bioreactor
Source: Sci Rep. 2022 Nov 23;12:20161. doi: 10.1038/s41598-022-24687-4 (PMC9686226; doi:10.1038/s41598-022-24687-4)
Supplement: Supplementary file 1 — Supplementary Information 1. [file 41598_2022_24687_MOESM1_ESM.zip › raw data/Transcriptomic raw data/6.Enrichment/2.KEGG/TE1vsTC1/UP/TE1vsTC1.up_KEGGbar.pdf]

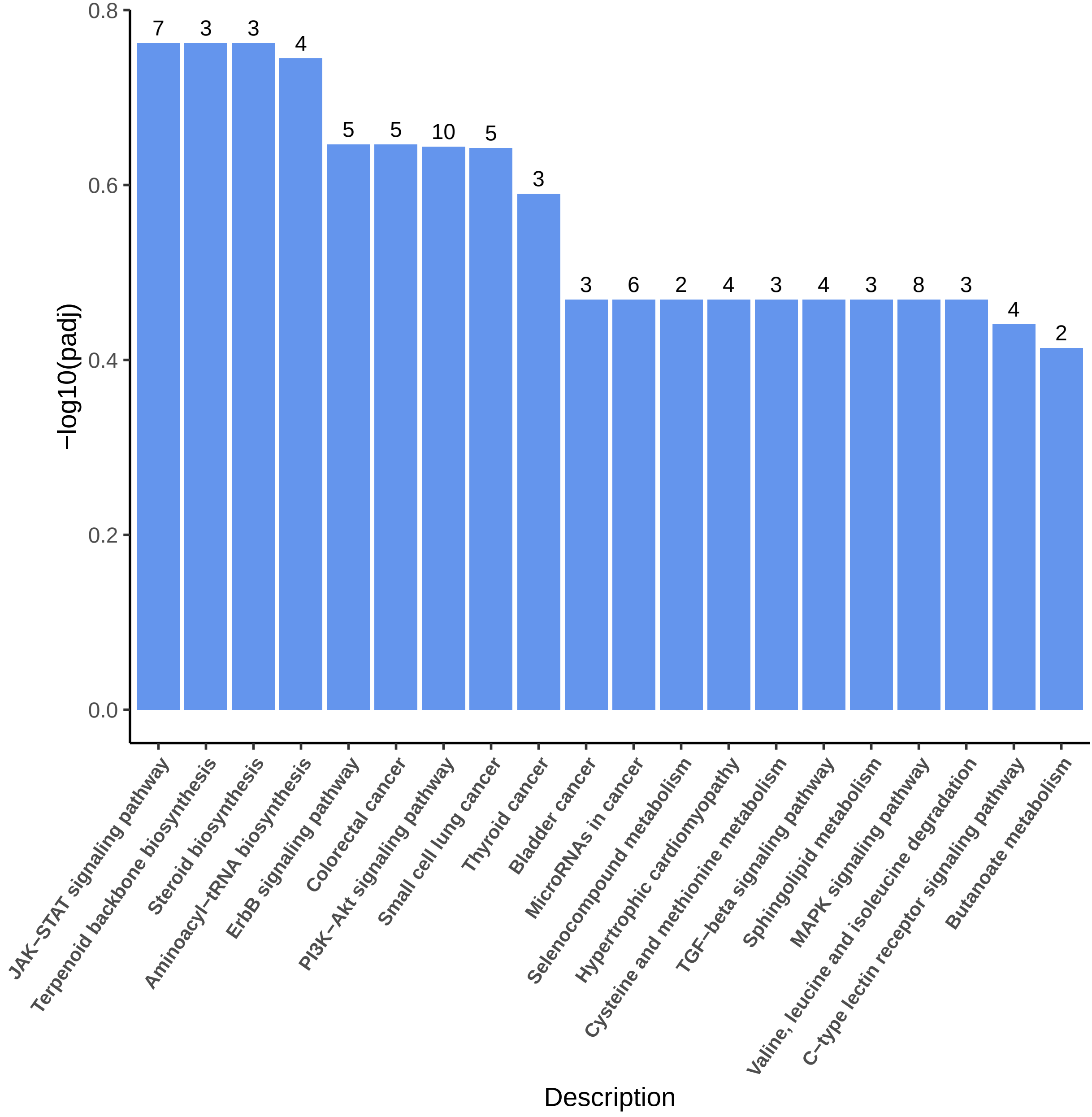

−log10(padj)
0.0
0.2
0.4
0.6
0.8
7
3
3
4
5
5
10
5
3
3
6
2
4
3
4
3
8
3
4
2
JAK−STAT signaling pathway
Terpenoid backbone biosynthesis
Steroid biosynthesis
Aminoacyl−tRNA biosynthesis
ErbB signaling pathway
Colorectal cancer
PI3K−Akt signaling pathway
Small cell lung cancer
Thyroid cancer
Bladder cancer
MicroRNAs in cancer
Selenocompound metabolism
Hypertrophic cardiomyopathy
Cysteine and methionine metabolism
TGF−beta signaling pathway
Sphingolipid metabolism
MAPK signaling pathway
Valine, leucine and isoleucine degradation
C−type lectin receptor signaling pathway
Butanoate metabolism
Description
